# Contrast medium administration with a body surface area protocol in step-and-shoot coronary computed tomography angiography with dual-source scanners

**DOI:** 10.1038/s41598-020-73915-2

**Published:** 2020-10-07

**Authors:** Liang Jin, Yiyi Gao, Yingli Sun, Cheng Li, Pan Gao, Wei Zhao, Ming Li

**Affiliations:** 1 Radiology Department, Huadong Hospital, Affiliated to Fudan University, Shanghai, China; 2 Institute of Functional and Molecular Medical Imaging, Fudan University, Shanghai, China

**Keywords:** Radiography, Tomography

## Abstract

We evaluated the feasibility and image quality of prospective electrocardiography (ECG)-triggered coronary computed tomography angiography (CCTA) using a body surface area (BSA) protocol for contrast-medium (CM) administration on both second- and third-generation scanners (Flash and Force CT), without using heart rate control. One-hundred-and-eighty patients with suspected coronary heart disease undergoing CCTA were divided into groups A (BSA protocol for CM on Flash CT), B (body mass index (BMI)-matched patients; BMI protocol for CM on Flash CT), and C (BMI-matched patients; BSA protocol for CM on Force CT). Patient characteristics, quantitative and qualitative measures, and radiation dose were compared between groups A and B, and A and C. Of the 180 patients, 99 were male (median age, 62 years). Average BSA in groups A, B, and C was 1.80 ± 0.17 m^2^, 1.74 ± 0.16 m^2^, and 1.64 ± 0.17 m^2^, respectively, with groups A and C differing significantly (*P* < 0.001). Contrast volume (50.50 ± 8.57 mL vs. 45.00 ± 6.18 mL) and injection rate (3.90 ± 0.44 mL/s vs. 3.63 ± 0.22 mL/s) differed significantly between groups A and C (*P* < 0.001). Groups A and C (both: all CT values > 250 HU, average scores > 4) achieved slightly lower diagnostic image quality than group B. The BSA protocol for CM administration was feasible in both Flash and Force CT, and therefore may be valuable in clinical practice.

## Introduction

In 2018, coronary heart disease (CHD) was the leading cause of death in the United States, accounting for 43.8% of deaths overall^1^. Coronary computed tomography angiography (CCTA) is a noninvasive technique that plays an important role in CHD screening and diagnosis^2–5^. The use of iodinated contrast medium (CM) provides sufficient vessel attenuation to allow proper evaluation of blood vessel lesions^6,7^. However, with the increase in the number of examinations, the use of iodinated CM for CCTA has become a concern, as it may lead to contrast-induced nephropathy (CIN)^8–11^, and CM remaining in the right cardiac cavity after the scan is not useful. Although the relationship between CCTA and the development of CIN is under debate, elderly patients with cardiac disease are considered at risk of developing CIN^9,12^. With the appearance of CT scanners with faster gantry rotation (≤ 350 ms) and wider coverage, scan duration is now shorter, which allows adjustment of the protocol for CM administration^9,10,13–16^.

Most protocols for CM administration are based on body mass index (BMI) or body weight^7,8,14,17,18^. Some more recent protocols have been based on the patient’s blood volume^9^ or use a high delivery rate^13^. However, the enhancement of vessel segments is influenced by the patient’s weight, height (expressed in terms of BMI and body surface area [BSA]), and cardiac output^19–22^.

BSA, an index that is widely used in clinical practice, has been identified as the most promising parameter for adjusting the contrast bolus in future protocols; indeed, an increase in blood volume is well paralleled by BSA^21,22^. In fact, BSA is considered a better indicator of metabolic mass than body weight, because the former is less affected by abnormal adipose mass^21,22^. Although there is currently no standard BSA protocol for CM administration, a previous study reported a BSA-adapted scanning protocol in prospective electrocardiography (ECG)-triggered sequence acquisition mode (step-and-shoot) CCTA, with a 64-slice scanner (heart rate < 65 bpm)^21^. However, the feasibility of using a BSA-based protocol for CM administration without heart rate control on dual-source CT (DSCT) has not been studied. Similarly, it is unknown whether the BSA protocol can produce satisfactory image quality at a lower CM volume and injection rate in DSCT.

Therefore, in this study, we aimed to establish whether using a BSA protocol for CM administration, which involved using less CM, and adjusting the injection rate, was feasible in step-and-shoot CCTA. To this end, we first compared the image quality of the three main coronary arteries visualized with a second-generation DSCT scanner, using a BSA protocol for CM administration in step-and-shoot CCTA, without heart rate control, with that of a BMI protocol. Second, we compared the image quality obtained with this BSA protocol between second-generation and third-generation DSCT scanners.

## Materials and methods

### Patients

One-hundred-and-eighty patients with suspected coronary heart disease who were scheduled for CCTA examination between January 2018 and January 2019 were enrolled. The 180 patients were divided into three groups: 60 patients underwent step-and-shoot CCTA on a second-generation DSCT scanner, with a BSA protocol for CM administration^21^ (group A; Table 1); 60 BMI-matched patients underwent step-and-shoot CCTA on a second-generation DSCT scanner with a BMI protocol for CM administration^23^ (group B, reference group; Table 1); and 60 BMI-matched patients underwent step-and-shoot CCTA on a third-generation DSCT scanner with a BSA protocol for CM administration (group C; Table 1). BSA (m^2^) was obtained using Stevenson’s formula (BSA [m^2^] = 0.0061 × height [cm] + 0.0128 × weight [kg] − 0.1529) for Chinese adults^24,25^

**Table 1:** Body surface area (BSA)-adapted and body mass index (BMI)-adapted contrast-medium injection protocol.

BSA				BMI			
BSA (m^2^)	Contrast volume (mL)	Saline volume (mL)	Flow rate (mL/s)	BMI (kg/m^2^)	Contrast volume (mL)	Saline volume (mL)	Flow rate (mL/s)
≤ 1.70	40	50	3.5	≤ 20.0	45	50	4
1.70–1.79	45	50	3.5	20.1–24.9	50	45	4
1.80–1.94	55	40	4.0	25.0–29.9	55	40	4
1.95–2.14	60	35	4.5	≥ 30.0	60	35	5
> 2.15	70	20	5.0				

The exclusion criteria were as follows: (1) patients allergic to iodine contrast agent or with severe renal insufficiency (creatinine ≤ 120 μmol/L); (2) patients with decompensated cardiac insufficiency; (3) patients taking drugs to control heart rate before examination; and (4) patients with arrhythmia, who could not hold their breath, or who had undergone stent implantation or coronary artery bypass grafting.

This prospective study was approved by the ethics committee of Huadong hospital (2019K005) and was carried out in accordance with relevant guidelines and regulations with ‘Discussion and evaluation of optimal use of contrast medium in coronary CT angiography’. All patients signed an informed consent form.

### Image acquisition and reconstruction

A second-generation DSCT scanner (Somatom Definition Flash, Siemens Healthcare, Forchheim, Germany) was used in groups A and B, while a third-generation DSCT scanner (Somatom Force, Siemens Healthcare) was used in group C. In all groups, the slice thickness and the interval of image reconstruction were 0.75 mm (see Supplementary Material 1, Tables 1, 2).

**Table 2:** Body mass index (BMI)-adapted scanning parameters.

BMI		
BMI (kg/m^2^)	Voltage (kV)	Current (mA)
≤ 20.0	70	CARE dose (4D)
20.1–24.9	80	CARE dose (4D)
25.0–27.4	100	250
27.5–29.9	100	280
≥ 30.0	120	300

### Quantitative and qualitative evaluation

The CT values and standard deviations (SDs) of coronary arteries were measured and as a qualitative analysis, double-blinded subjective scoring of image quality was performed (see Supplementary Material 2).

### Radiation dose

Only the CCTA scanning dose was counted, and the scout view, coronary artery calcium score, and the radiation dose of the automatic bolus-tracking technique were not included. Dose length product (DLP) was automatically determined by the CT scanner. Effective radiation dose (ED) was estimated by multiplying the DLP by a conversion factor of 0.014 mSv/(mGy × cm)^13,14^.

### Statistical analysis

IBM SPSS Statistics 22 (IBM, Chicago, IL, USA) software was used for statistical analysis. Levene’s test was used to assess normality of distribution of continuous variables. Continuous variables were expressed as means ± SD. Differences in patient characteristics, radiation dose, and quantitative and qualitative measures between groups A and B as well as between groups A and C were tested for significance with the independent-samples *t*-test. A two-tailed *P*-value (*P*) < 0.05 was considered to be statistically significant. Kappa analysis was used to evaluate interobserver agreement. The kappa value was defined as follows: < 0.20, poor agreement; 0.21–0.40, slight agreement; 0.41–0.60, moderate agreement; 0.61–0.80, good agreement; and 0.81–1.00, almost perfect agreement^26^.

## Results

CCTA was successfully performed in all 180 patients (99 [55%] men and 81 [45%] women; median age, 62 years). The patient characteristics and radiation doses are shown in Table 3.

**Table 3:** Patient characteristics and radiation dose comparison.

Parameters	Group A (N = 60)	Group B (N = 60)	*P* value	Group A (N = 60)	Group C (N = 60)	*P* value
BSA (m^2^)	1.80 ± 0.17	1.74 ± 0.16	0.061	1.80 ± 0.17	1.64 ± 0.17	0.0001
BMI (kg/m^2^)	24.75 ± 2.57	23.96 ± 2.35	0.083	24.75 ± 2.57	24.10 ± 2.68	0.184
CM (mL)	50.50 ± 8.57	51.00 ± 2.02	0.662	50.50 ± 8.57	45.00 ± 6.18	0.0001
FL (mL/s)	3.90 ± 0.44	4.00 ± 0.00	0.083	3.90 ± 0.44	3.63 ± 0.22	0.0001
DLP	190.39 ± 97.21	184.88 ± 64.20	0.715	190.39 ± 97.21	196.57 ± 51.91	0.665
ED (mSv)	2.66 ± 1.36	2.59 ± 0.89	0.715	2.66 ± 1.36	2.75 ± 0.73	0.665
HR (bpm)	64.88 ± 11.03 (39–94)	64.67 ± 13.17 (41–90)	0.924	64.88 ± 11.03 (39–94)	61.70 ± 12.19 (65–90)	0.137

BMI, body mass index; BSA, body surface area; CM, contrast medium; DLP, dose length product; ED, effective radiation dose; FL, flow rate; HR, heart rate.

### Quantitative and qualitative evaluation

Table 4 shows a comparison of the measured CT values and subjective image quality scores (see Supplementary Material 3, Fig. 1). Representative images obtained with the Flash and Force CT scanners are shown in Figs. 2 and 3, respectively.

**Table 4:** Comparison of quantitative and qualitative analysis results.

Parameters	Group A (N = 60)	Group B (N = 60)	*P* value	Group A (N = 60)	Group C (N = 60)	*P* value
AO (HU)	469.80 ± 97.69	566.78 ± 102.23	0.0001	469.80 ± 97.69	505.63 ± 111.56	0.064
LAD-P (HU)	474.23 ± 108.96	578.22 ± 97.30	0.0001	474.23 ± 108.96	476.17 ± 113.35	0.925
LAD-D (HU)	300.19 ± 76.61	332.53 ± 113.07	0.070	300.19 ± 76.61	256.18 ± 68.75	0.0001
LCX-P (HU)	474.23 ± 108.96	578.22 ± 97.30	0.0001	474.23 ± 108.96	476.17 ± 113.35	0.925
LCX-D (HU)	344.08 ± 108.00	379.08 ± 93.99	0.061	344.08 ± 108.00	283.48 ± 74.33	0.001
RCA-P (HU)	469.12 ± 93.98	545.12 ± 91.12	0.0001	469.12 ± 93.98	485.95 ± 192.32	0.544
RCA-D (HU)	415.35 ± 100.27	473.24 ± 110.85	0.003	415.35 ± 100.27	469.86 ± 142.09	0.017
AO_CNR	14.83 ± 22.69	11.60 ± 6.87	0.293	14.83 ± 22.69	8.87 ± 5.62	0.052
LAD-P_CNR	39.51 ± 16.88	51.56 ± 30.59	0.009	39.51 ± 16.88	35.32 ± 18.50	0.198
LCX-P_CNR	39.51 ± 16.88	51.56 ± 30.59	0.009	39.51 ± 16.88	35.32 ± 18.50	0.198
RCA-P_CNR	39.42 ± 17.35	47.78 ± 26.85	0.045	474.23 ± 108.96	34.89 ± 17.31	0.155
Qualitative analysis for RCA	4.62 ± 0.53 (3.5–5)	4.72 ± 0.36 (4–5)	0.231	4.62 ± 0.53 (3.5–5)	4.83 ± 0.33 (4–5)	0.011
Qualitative analysis for LAD	4.16 ± 0.56 (3–5)	4.40 ± 0.39 (3.5–5)	0.016	4.16 ± 0.56 (3–5)	4.40 ± 0.34 (4–5)	0.012
Qualitative analysis for LCX	4.17 ± 0.60 (3–5)	4.25 ± 0.53 (3–5)	0.423	4.17 ± 0.60 (3–5)	4.15 ± 0.32 (3.5–5)	0.850

AO, aortic root; HU, Hounsfield units; LAD-D, distal left anterior descending; LAD-P, proximal left anterior descending; LCX-D, distal left circumflex; LCX-P, proximal left circumflex; PVAT, perivascular adipose tissue; RCA-D, distal proximal right coronary artery; RCA-P, proximal right coronary artery.

**Figure 1:**
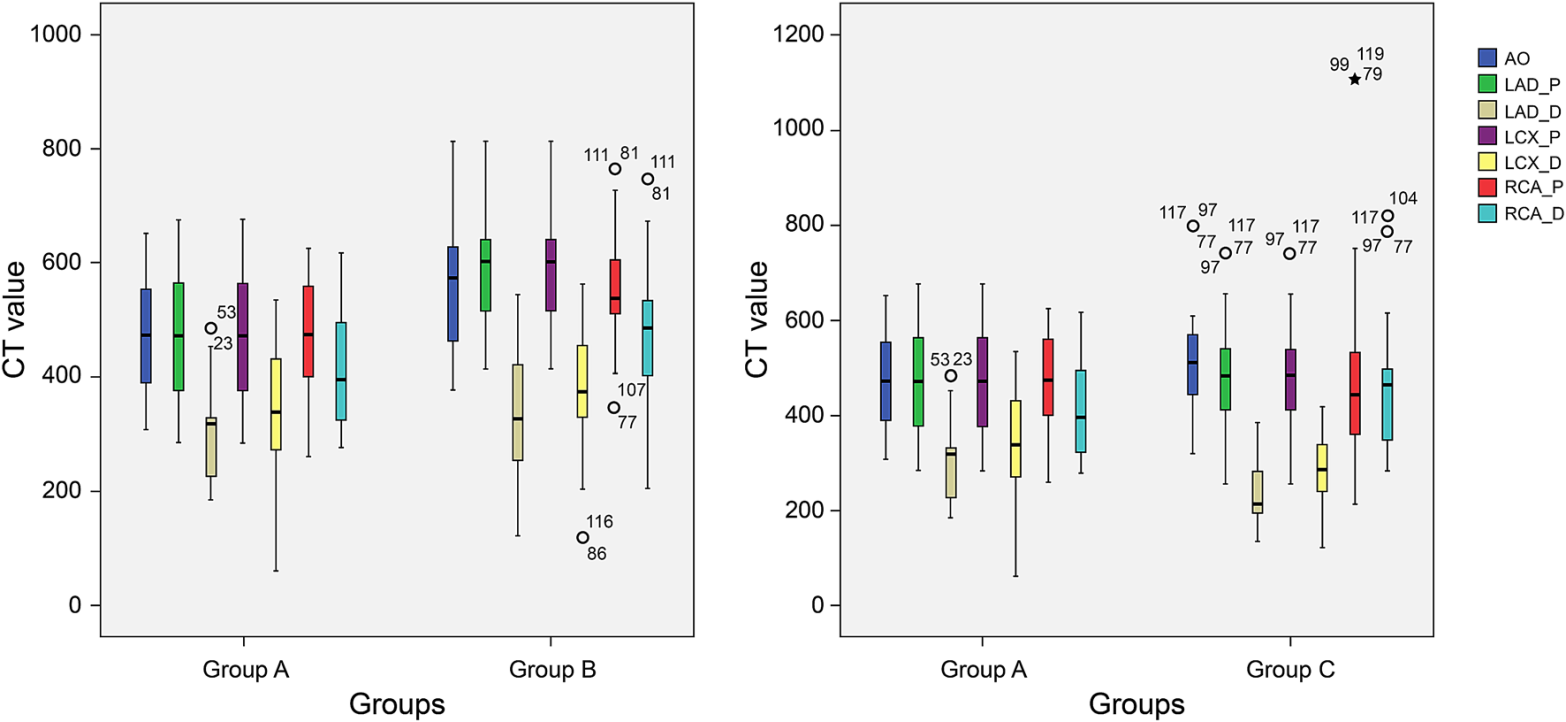
Comparison of CT values of all measurements in all groups. (**A**) Comparison of groups A and B. (**B**) Comparison of groups A and C.

**Figure 2:**
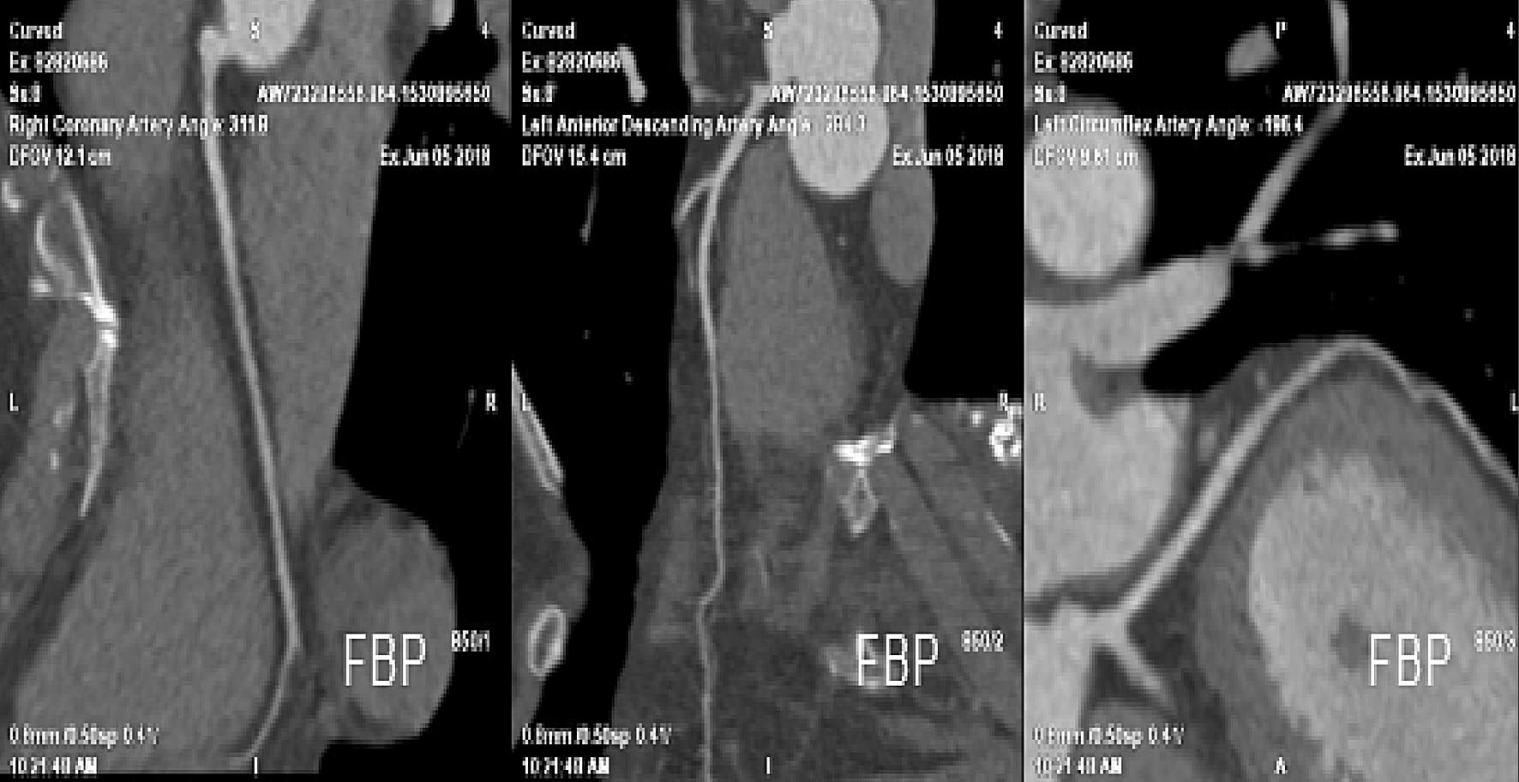
Representative image of a 70-year-old female patient, body surface area (BSA) 1.58 m^2^, body mass index (BMI) 27.27 kg/m^2^, heart rate (HR) 96 bpm, obtained using 40 mL contrast agent injected at a flow rate of 3.5 mL/s on a Flash computed tomography scanner.

**Figure 3:**
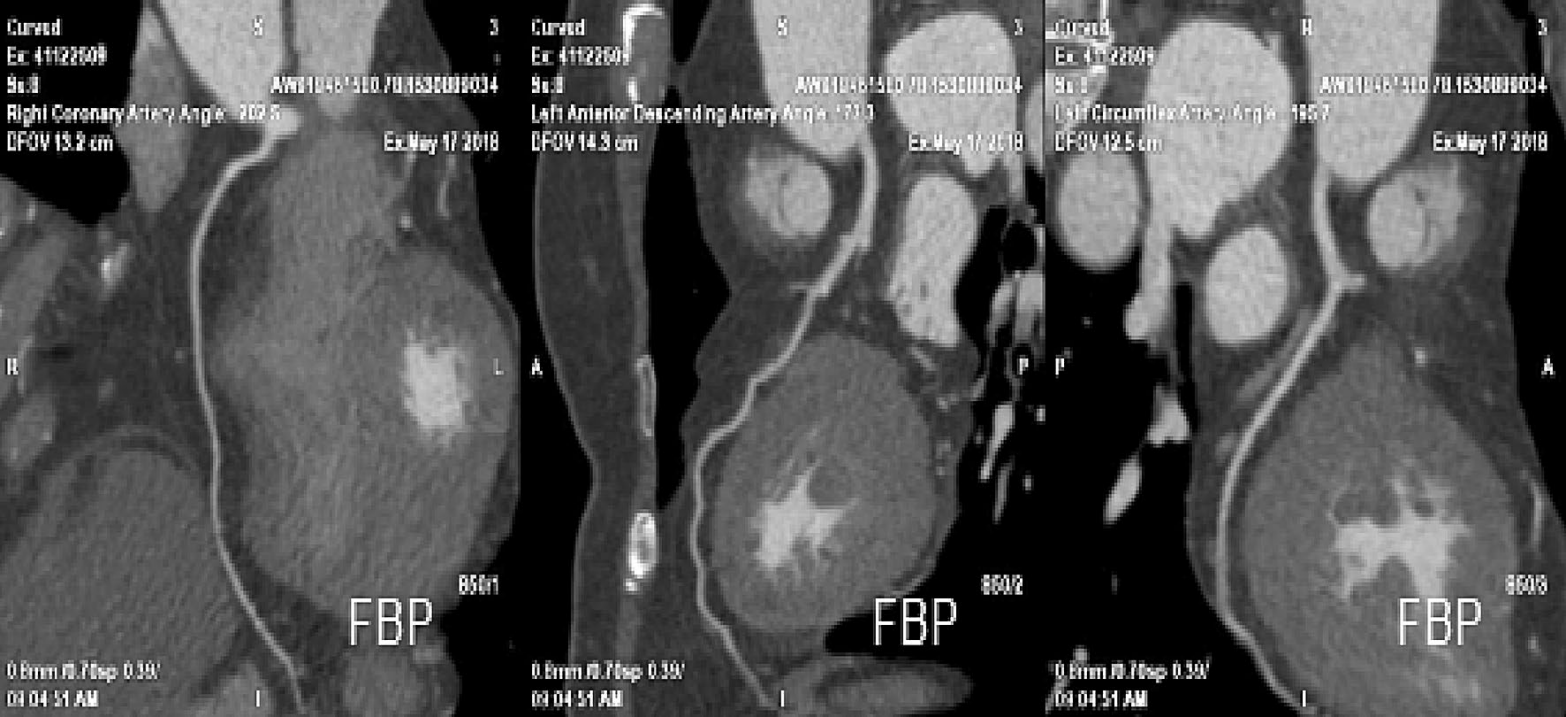
Representative image of a 59-year-old female patient, body surface area (BSA) 1.48 m^2^, body mass index (BMI) 23.8 kg/m^2^, heart rate (HR) 85 bpm, using 40 mL of contrast agent injected at a flow rate of 3.5 mL/s on a Force computed tomography scanner.

## Discussion

In this study, we compared the feasibility of using a BSA protocol for CM administration in step-and-shoot CCTA on Flash and Force CT; implementation of the protocol was feasible, and the image quality obtained on both second- and third-generation CT scanners was satisfactory for diagnosis.

Pazhenkottil et al. demonstrated that a CM-injection protocol based on BSA was feasible with a 64-slice CT scanner, using a step-and-shoot acquisition mode, in 2010^21^. However, in daily practice, we found that the BSA-adapted scanning protocol was not suitable for use in DSCT with free heart rate, as some CM remained in the right heart chambers after the scan. CIN is closely related to existing renal insufficiency and the use of a large amount of CM^27,28^.

With both scanners used in this study, the scan duration is shorter, reducing the amount of CM required. The Flash CT scanner is a 64-slice scanner with two X-ray tubes, while the Force CT scanner has an additional wider-coverage detector (5.76 cm, 96-slice)^29,30^. Hence, in this study, we designed a new BSA protocol for Chinese adults in which the maximum contrast volume was reduced to 90 mL, corresponding to a maximum injection rate of 5 mL/s to keep the injection duration similar (approximately 13 s) for a BSA of 1.7–2.14 m^2^, with a reduction of 20 mL of contrast volume for large BSAs (> 2.15 m^2^).

A recent study used a first-generation DSCT scanner with a double low-dose strategy and a high iodine delivery rate (IDR) of more than 2.0 g iodine/s in terms of the total iodine dose (TID)^13^, and achieved a TID of 19.5 ± 2.7 g iodine (gI) with an IDR of 2.22 gI/s. In our study, we achieved a TID of 18.69 ± 3.17 gI with an IDR of 1.44 ± 0.16 gI/s in group A. Our values were reduced (TID: 4%; IDR: 35%) without a decrease in diagnostic image quality. The BMI, BSA, and iodine concentration of group A were similar to those of group 2 in the previous study^13^. In the present study, group A had an average volume of 50.50 ± 8.57 mL (range, 40–70 mL) and average BSA of 1.80 ± 0.17 m^2^ (approximate TID per m^2^, 10.23 gI/m^2^) while group C had an average volume of 45.00 ± 6.18 mL (range, 40–55 mL) and average BSA of 1.64 ± 0.17 m^2^ (approximately 10.15 gI/m^2^). This corresponded to a reduction of 19% and 20.5% in TID/m^2^, respectively, as compared with the 70.9 ± 14.1 mL for 1.98 m^2^ (approximately 12.77 gI/m^2^) in a study by Pazhenkottil et al^21^.

In quantitative and qualitative analysis of Flash CT images, group A showed an average CT value of more than 300 HU in all measured segments. Although vessel enhancement was slightly lower than that in group B (the reference group), and the average qualitative scores in group B were better than those in group A, the image quality was not affected, as the optimal images had high intra-arterial opacification of more than 250 HU^11^. Moreover, all qualitative scores exceeded 4. The BSAs of groups A and C were significantly different (*P* < 0.001), which resulted in significant differences in both contrast volume and injection rate (*P* < 0.001). Nevertheless, the image quality met diagnostic demands, with subjective scores > 4. Furthermore, vessel enhancement in the AO and proximal coronary arteries was greater than 400 HU, and even the lower levels of enhancement in the distal coronary arteries exceeded 250 HU.

Heart rate is one of the main factors affecting the quality of coronary imaging^31–35^, determining the acquisition mode of CCTA. Previous studies have suggested that high-pitch scanning was helpful to reduce the contrast-medium volume and injection rate^10,17,36^, due to the very short scan duration (< 1 s). However, high-pitch scanning is strictly limited by heart rate. Gordic et al. proved that the diagnostic rate in cases with a heart rate > 75 bpm on Force CT was only 14%. In fact, using a high-pitch mode on Flash CT requires that the heart rate did not exceed 63 bpm; this requirement was relaxed to 70 bpm on Force CT^29^. Step-and-shoot acquisition has advantages over high-pitch scanning, due to the more relaxed heart rate limits^9,16,22^. as well as over retrospective ECG-gated scanning, due to its lower radiation dose^37–39]^. However, it involves a longer acquisition time for a higher injection rate to maintain sufficient peak intravascular enhancement. In this study, heart rate ranged from 39 bpm to 94 bmp. Higher heart rates were mainly supported by the fast gantry rotation of DSCT, and the temporal resolution was increased from 83 to 75 ms for the Flash CT and to 66 ms for the Force CT. A previous study reported decreased coronary arterial attenuation with an increased heart rate during DSCT-CCTA, without heart rate control during data acquisition^6^. Our findings demonstrated the feasibility of using a BSA-based protocol with a lower contrast-medium volume and a slower injection rate at higher heart rates. This was consistent with the findings of a previous study^21^ that showed that lower injection rates (< 5 mL/s) were sufficient for coronary artery enhancement in step-and-shoot acquisition (when the heart rate < 65 bpm).

The present study had some limitations. First, the study covered relatively narrow BMI and BSA ranges. Therefore, assuming potential differences in body composition extremes, it may not be possible to generalize or extrapolate our findings to other populations. Second, further improvement in terms of even lower CM volume and slower injection rate is possible, because CM was still found in the right atrium after scanning in some patients, which could decrease image quality. Third, coronary angiography was not considered as the gold standard. Fourth, although the BSA of group C was not matched, it was still challenging to obtain sufficient enhancement in vessel segments with the lower contrast volume and slower injection rate in cases with higher heart rates and the image quality in group C was also satisfied. Therefore, the image quality could not be compared between groups. Nevertheless, the image quality still indicated the feasibility of using the BSA protocol on Force CT.

## Conclusion

Using the BSA protocol for CM administration, we could achieve better diagnostic image quality in step-and-shoot CCTA with free heart rate than with the BMI protocol, on both Flash and Force CT scanners. Thus, we demonstrated that using the BSA protocol for CM administration was feasible in step-and-shoot CCTA.

## Supplementary information

Supplementary Information 1.

Supplementary Information 2.

Supplementary Information 3.

## Abbreviations

AO: Aortic root
BMI: Body mass index
BSA: Body surface area
CCTA: Coronary computed tomography angiography
CHD: Coronary heart disease
CIN: Contrast-induced nephropathy
CM: Contrast-medium
CT: Computed tomography
DSCT: Dual-source computed tomography
ECG: Electrocardiography
LAD-D: Distal left anterior descending
LAD-P: Proximal left anterior descending
LCX-D: Distal left circumflex
LCX-P: Proximal left circumflex
PVAT: Perivascular adipose tissue
RCA-D: Distal proximal right coronary artery
RCA-P: Proximal right coronary artery
