# Supplementary material for: Contrast medium administration with a body surface area protocol in step-and-shoot coronary computed tomography angiography with dual-source scanners
Source: Sci Rep. 2020 Oct 7;10:16690. doi: 10.1038/s41598-020-73915-2 (PMC7541528; doi:10.1038/s41598-020-73915-2)
Supplement: Supplementary file 1 — Supplementary Information 1. [file 41598_2020_73915_MOESM1_ESM.docx]

**Contrast medium administration with a body surface area protocol in step-and-shoot coronary computed tomography angiography with dual-source scanners**

Liang Jin^a,1^ MD, Yiyi Gao^a,1^ MD, Yingli Sun^a^ MD, Cheng Li^a^ MD, Pan Gao^a^ MD, Wei Zhao^a^ PhD, Ming Li^a,b,*^ PhD

**Supplementary Material 1**

*Image acquisition and reconstruction*

A second-generation DSCT scanner (Somatom Definition Flash, Siemens Healthcare, Forchheim, Germany) was used in groups A and B, with collimation 2 × 64 × 0.6 mm and rotation time 0.28 s/rotation. A third-generation DSCT scanner (Somatom Force, Siemens Healthcare) was used in group C, with collimation 2 × 96 × 0.6 mm and rotation time 0.25 s/rot. In all groups, the scanning range started from the lower part of the protuberance of the tracheal bifurcation and proceeded in a cranio-caudal direction to the diaphragmatic surface of the heart. A prospective ECG-triggered sequence acquisition mode (step-and-shoot) was used. The tube current and tube voltage used are shown in Table 1-B. The automated anatomical tube current modulation technique (CARE Dose 4D, Siemens Healthcare) was implemented, and the range of exposure dose (ECG pulsing) was 35%–80% in the R–R interval. As CM, iopamidol (370 mg iodine [mgI]/mL) was injected using a 20-G closed intravenous catheter system with an Ulrich high-pressure syringe. The bolus tracking technique was used for threshold monitoring at the aortic root (AO), with an enhancement threshold of 80 HU and a delay time of 7 s.

In all groups, the slice thickness and the interval of image reconstruction were 0.75 mm. The kernel was B26f and Bv36 in Flash and Force scanners, respectively. The optimal phase (end-systolic or end-diastolic) of coronary artery display was automatically reconstructed by the scanner using the FBP algorithm. All reconstructed images were then transferred to the post-processing workstation (Syngo MMW\,P VE 36A, Siemens Healthcare), and axial images were used for analysis.

Table 1-A. Body surface area (BSA)-adapted and body mass index (BMI)-adapted contrast-medium injection protocol

| BSA | | | | BMI | | | |
| --- | --- | --- | --- | --- | --- | --- | --- |
| BSA (m^2^) | Contrast volume (mL) | Saline volume(mL) | Flow rate (mL/s) | BMI (kg/m^2^) | Contrast volume (mL) | Saline volume (mL) | Flow rate (mL/s) |
| ≤ 1.70 | 40 | 50 | 3.5 | ≤ 20.0 | 45 | 50 | 4 |
| 1.70–1.79 | 45 | 50 | 3.5 | 20.1–24.9 | 50 | 45 | 4 |
| 1.80–1.94 | 55 | 40 | 4.0 | 25.0–29.9 | 55 | 40 | 4 |
| 1.95–2.14 | 60 | 35 | 4.5 | ≥ 30.0 | 60 | 35 | 5 |
| >2.15 | 70 | 20 | 5.0 |  |  |  |  |

Table 1-B. Body mass index (BMI)-adapted scanning parameters

| BMI | | |
| --- | --- | --- |
| BMI (kg/m^2^) | Voltage (kV) | Current (mA) |
|  |  |  |
| ≤ 20.0 | 70 | CARE Dose (4D) |
| 20.1–24.9 | 80 | CARE Dose (4D) |
| 25.0–27.4 | 100 | 250 |
| 27.5–29.9 | 100 | 280 |
| ≥ 30.0 | 120 | 300 |
