# Supplementary material for: Contrast medium administration with a body surface area protocol in step-and-shoot coronary computed tomography angiography with dual-source scanners
Source: Sci Rep. 2020 Oct 7;10:16690. doi: 10.1038/s41598-020-73915-2 (PMC7541528; doi:10.1038/s41598-020-73915-2)
Supplement: Supplementary file 2 — Supplementary Information 2. [file 41598_2020_73915_MOESM2_ESM.docx]

**Contrast medium administration with a body surface area protocol in step-and-shoot coronary computed tomography angiography with dual-source scanners**

Liang Jin^a,1^ MD, Yiyi Gao^a,1^ MD, Yingli Sun^a^ MD, Cheng Li^a^ MD, Pan Gao^a^ MD, Wei Zhao^a^ PhD, Ming Li^a,b,*^ PhD

**Supplementary Material 2**

*Quantitative and qualitative evaluation*

The CT values and standard deviations (SDs) of the lumens of the aortic root (AO), proximal left anterior descending (LAD-P) artery, distal left anterior descending (LAD-D) artery, proximal left circumflex (LCX-P) artery, distal left circumflex (LCX-D) artery, proximal right coronary artery (RCA-P), and distal proximal right coronary artery (RCA-D), and of the perivascular adipose tissue (PVAT) were measured by a radiologist (P.G., with 5 years of experience in cardiovascular diagnosis). The region-of-interest (ROI) of the AO was set to 90 mm^2^, while the ROIs for the other arteries were set to 1 mm^2^. The image noise was determined as the SD of attenuation at the ROI in the AO. The contrast noise ratio (CNR) was calculated as (CT value-CT value of PVAT)/SD of PVAT.

As a qualitative analysis, double-blinded subjective scoring of image quality was performed by two experienced radiologists (Y.G. with 8 years, and W.W. with > 15 years, of experience in cardiovascular diagnosis). The segmentation standard of 15 segments (excluding the intermediate branch) proposed by the American Heart Association in 1975 was adopted.

The scoring standard of the 5-point Likert scale used for image interpretation was as follows: 1 = poor opacification, insufficient for diagnosis; 2 = suboptimal opacification, low diagnostic confidence; 3 = acceptable opacification, sufficient for diagnosis; 4 = good opacification of proximal and distal segments; and 5 = excellent opacification of proximal and distal segments.(13) If consistency between the two readers was not good (4 on the 5 point scale), a third reader (M.L. with > 20 years of experience in cardiovascular diagnosis) interpreted the images; otherwise, the interpretation of the more experienced reader (W.W.) was used. The readers were allowed to adjust the window settings freely.
