# Supplementary material for: Contrast medium administration with a body surface area protocol in step-and-shoot coronary computed tomography angiography with dual-source scanners
Source: Sci Rep. 2020 Oct 7;10:16690. doi: 10.1038/s41598-020-73915-2 (PMC7541528; doi:10.1038/s41598-020-73915-2)
Supplement: Supplementary file 3 — Supplementary Information 3. [file 41598_2020_73915_MOESM3_ESM.docx]

**Contrast medium administration with a body surface area protocol in step-and-shoot coronary computed tomography angiography with dual-source scanners**

Liang Jin^a,1^ MD, Yiyi Gao^a,1^ MD, Yingli Sun^a^ MD, Cheng Li^a^ MD, Pan Gao^a^ MD, Wei Zhao^a^ PhD, Ming Li^a,b,*^ PhD

**Supplementary Material 3**

*Quantitative and qualitative evaluation*

Table 3 shows the comparison of the measured CT values and the subjective image quality scores. On Flash CT, CT values of AO, LAD-P, LCX-P, RCA-P, and RCA-D in group A were significantly lower than those in group B, while the LAD_P_CNR, LCX_P_CNR, and RCA_P_CNR also differed significantly between the groups. On Force CT, only the CT values of LAD-D, LCX-D, and RCA-D of groups A and C differed significantly while the CNRS values were similar (Figure 1).

Kappa analysis revealed good agreement of the subjective scores between the two observers (kappa = 0.86); therefore, the results of only a single observer (W.W.) were used for further analysis. Qualitative scores for the LAD artery in group A (4.16 ± 0.56) was significantly lower than that in group B (4.40 ± 0.39). Qualitative scores for the RCA and LAD artery in group A (4.62 ± 0.53 and 4.16 ± 0.56, respectively) were significantly lower than those in group C (4.83 ± 0.33 and 4.40 ± 0.34, respectively). Qualitative analysis indicated that the image quality of all CCTAs satisfied the diagnostic demand (average score > 4). Representative images obtained with the Flash and Force CT scanners are shown in Figures 2 and 3, respectively.
